# Referral to and Engagement with Pulmonary Rehabilitation: A Review and Strategic Behavioural Analysis of Factors Influencing Behaviour and Intervention Opportunities

**DOI:** 10.2147/COPD.S558338

**Published:** 2026-05-30

**Authors:** Amanda P Moore, Isolt Reardon, Osanna Ramkissoon, Frances Early, Estelle Payerne, Polly-Anna Ashford, Stephen Holmes, Michael P Kelly, Sally J Singh, G M Monsur Habib, Jonathan Fuld, Fabiana Lorencatto, On behalf of the UPTURN Team

**Affiliations:** 1 Centre for Behaviour Change, University College London, London, Greater London, UK; 2 Research and Development, Cambridge University Hospitals NHS Foundation Trust, Cambridge, Cambridgeshire, UK; 3 Norwich Clinical Trials Unit, University of East Anglia, Norwich, Norfolk, UK; 4 The Park Medical Practice, Shepton Mallet, Somerset, UK; 5 Department of Public Health and Primary Care, University of Cambridge, Cambridge, Cambridgeshire, UK; 6 Centre for Exercise and Rehabilitation Science, University of Leicester, University Hospitals Leicester NHS Trust, Leicester, Leicestershire, UK; 7 Mymensingh Medical College, The University of Edinburgh, Edinburgh, Midlothian, Scotland; 8 Victor Phillip Dahdaleh Heart and Lung Research Institute, Cambridge University Hospitals NHS Foundation Trust, Cambridge, Cambridgeshire, UK

**Keywords:** behaviour science, COPD, referral, pulmonary rehabilitation, intervention design

## Abstract

**Background:**

Despite strong evidence of benefit, in the UK only 40% of eligible patients are referred to Pulmonary Rehabilitation (PR) and there are poor levels of uptake and completion globally. Understanding how current interventions address reported barriers to PR referral and engagement can help understand intervention effectiveness and highlight opportunities for future policy and intervention.

**Methods:**

Two systematic reviews were conducted: 1) Review of studies reporting barriers/enablers to PR delivery and engagement behaviours, with extracted barriers/enablers inductively synthesized into themes and coded to the domains of the Theoretical Domains Framework (TDF). 2) Review of existing interventions to improve PR uptake/engagement, specifying component Behaviour Change Techniques (BCTs) using established taxonomies. Interventions were categorised for promise according to evidence of change in behaviour for one or more outcomes. Findings from both reviews were triangulated using the Theories and Techniques tool (TaTT), to assess the extent to which components in current interventions targeted key barriers/enablers.

**Results:**

Sixty-three studies were included in the analysis. Barriers and enablers to PR mainly fell under the TDF domains *Environment, context and resources; Knowledge; Social Influences; Professional role and identity*; and *Memory, attention and decision making*. There are opportunities to further support referral by considering interventions targeted at memory and decision making and by streamlining referral processes. For patient engagement with PR, the highly represented TDF domains were *Environment, context and resources; Social influences; Emotions; Knowledge*; and *Beliefs about capabilities*. Fifty-four percent of referral interventions and 15% of engagement interventions were considered very promising. BCTs in promising interventions targeting patient engagement included provision of social support and guidance on planning and goal setting. Opportunities for improving engagement include consideration of the emotional burden associated with COPD and attending PR.

**Conclusion:**

These findings can inform development of new, or refinement of existing, interventions targeting PR for people with COPD.

## Introduction

Chronic Obstructive Pulmonary Disease (COPD) is the third leading cause of death globally, accounting for more than 3 million deaths and 74 million disability-adjusted life years in 2019.^1^ Pulmonary rehabilitation (PR) is a structured, individually tailored, programme to support autonomy and self-management for people living with COPD.^2^ The programme includes exercise training, patient education, dietary education and behaviour change support.^3^ PR has been recommended as a highly effective part of COPD management.^4–7^ It reduces symptom severity, acute exacerbations and healthcare costs.^2^,^8^,^9^

Whilst there is overwhelming evidence of benefit, PR referral, engagement and adherence rates remain low in the UK and globally.^10–12^ In the UK, despite significant improvement over recent years, the recent Quality and Outcomes Framework (QOF) data shows only 40% of eligible patients receive a referral,^13^ echoing similar statistics in high income countries globally.^10^,^11^ Similarly, barriers to participation are indicated by poor uptake and completion rates.^14^ In the UK, around 30% of people referred do not attend.^15^ Barriers to participation are exacerbated amongst people from minority ethnicities and those facing other psychosocial and economic barriers to healthcare participation.^16^

Identifying influences on PR behaviours associated with referral and engagement is necessary to inform intervention design.^17^,^18^ To date, reviews of published studies indicate that organisational, relational, and individual-level influences can interact to influence PR referral and participation behaviours.^17^,^18^ However, much of this data is from before the recent pandemic. Post-pandemic reduction in COPD diagnosis rates and in access to fundamental respiratory care, including PR, have been reported, indicating a timely opportunity to review patient and HCP perspectives.^19^ Numerous interventions have been developed to date to target referral, engagement and ongoing COPD self-management.^17^,^20^,^21^ The systematic review conducted by Early et al suggested that significant increases in referral rates occurred in intervention studies that included components such as educational sessions for clinicians in primary care and collaborative learning sessions for HCPs.^17^,^22^,^23^ Effective interventions targeting PR delivery included tele-rehabilitation and home-PR.^24^,^25^ A recent study by Watson et al investigated the entire PR pathway by conducting a meta-analysis of 30 studies to identify the effectiveness of several PR interventions.^18^ This study found that interventions that included components such as staff learning activities, computer templates for COPD care, and multidisciplinary team meetings showed promising increases in PR referrals.^18^

Modifiable factors influencing behaviour, such as knowledge, beliefs, social and environmental factors, are potential targets of interventions to support behaviour change.^26^ Each stage of the PR pathway engages different behaviours and interventions targeting these elements, can be considered behaviour change interventions – strategically organised activities aimed at modifying specific behaviour patterns.^26^ Interventions are often developed with no formal examination of the target behaviour and are based on implicit common-sense models of behaviour.^26^,^27^ By applying a behaviour change perspective, researchers and healthcare providers can carefully dissect influences on complex behaviours and understand how interventions could be designed to best support behaviour change.^27^ Among previously published reviews, only Cox et al (2017) used a behavioural science-influenced theoretical framework to understand influences on referral and engagement behaviours No reviews to date have used a behavioural perspective to comprehensively and systematically evaluate PR interventions.^18^ Utilising a behaviour change lens can be instrumental in unravelling the effects of interventions and understanding the diverse factors that contribute to improved outcomes.^28^

**Figure 1:**
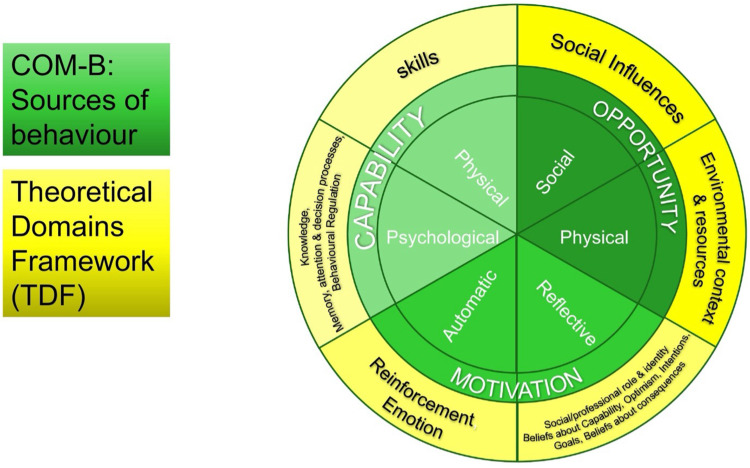
The COM-B model conceptualises behaviour as being a result of a person’s capability to perform the behaviour, their intention or motivation and opportunity factors in the social and physical environment that make the behaviour more or less likely. COM-B analysis supports systematic analysis of factors influencing a given behaviour. The TDF framework provides added granularity, to describe influences on behaviour which can be targeted in behaviour change interventions. The TDF domains can then be mapped to BCTs using the BCTTv1 and form the basis of the “mechanisms of action” or pathways through which BCTs take effect within an intervention.

**Figure 2:**
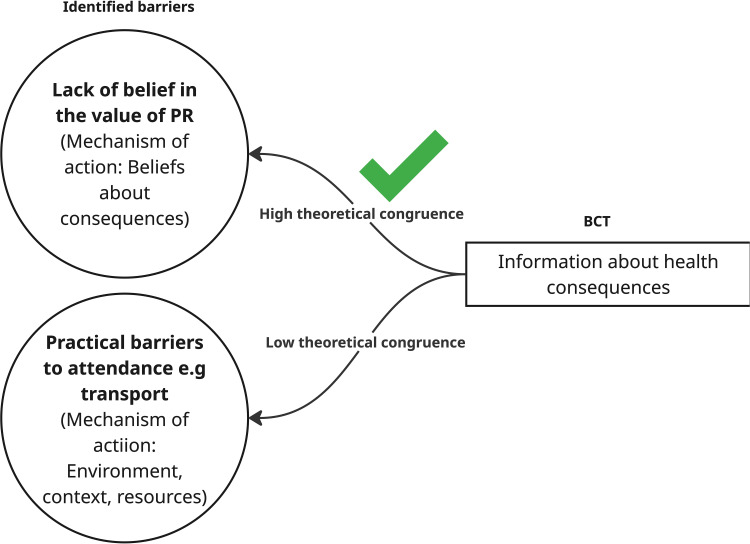
Illustration to show theoretical congruence.

Behavioural science frameworks such as the Behaviour Change Wheel (BCW) which includes the theoretical model of behaviour COM-B, The Theoretical Domains Framework (TDF) and the Behaviour Change Techniques Taxonomy (BCTTv1) can be used to help understand influences on behaviour and to describe intervention content (Figure 1). The TDF has been widely used to synthesise evidence of barriers and enablers in qualitative systematic reviews.^29^ Similarly, the BCW and BCTTv1 taxonomy are often used to specify and categorise BCTs in interventions and to explore the active ingredients associated with improved outcomes^30^ In this context, theoretical congruence represents a match between the BCT and the hypothesised mechanism of action (MoA).^29^ MoAs represent factors for which there is evidence of influence on behaviour change. For example, a theoretical congruence pairing would be using the *BCT Information about health consequences* to target the MoA Beliefs about health consequences (Figure 2). A lack of theoretical congruence would represent a mismatch, for example using the BCT *Information about health consequences* when the barrier is lack of available transport. Many interventions are not designed based on theory or the rationale for the design is not made explicit. This means that many interventions aiming to change behaviour lack a rationale for choosing an intervention approach and risk not being designed using BCTs targeting the factors that have been shown to influence a specific behaviour. Strategic behavioural analysis is an opportunity to assess this by comparing the theoretical congruence between BCTs used in an intervention against known barriers/enablers to a behaviour^31^ This enables us to identify areas of match (congruence) and mismatch (lack of congruence) in existing interventions as well as identifying opportunities for future interventions target barriers and enablers not currently considered in interventions as well as identifying theoretically congruent BCTs not currently use in interventions (missed opportunities). The empirically based Theories and Techniques Tool (TaTT) supports assessment of theoretical congruence by summarising the evidence linking BCTs to their domains of influence or mechanism of action, as specified in the TDF.^32^ The tool was developed from a literature synthesis^33^ and an expert consensus study.^34^ Exploring theoretical congruence, within the current intervention evidence enables elucidation of theoretical reasons for effectiveness as well as identification of opportunities for further intervention development and systems-level change.

Given the changes in the post-COVID-19 healthcare context, since the previous review by Cox et al in 2017, there is opportunity for a timely review of recent evidence of factors influencing PR related behaviours. Additionally, a systematic, theoretical analysis of existing interventions from a behavioural change perspective is needed.^17^ Understanding the core components of existing interventions targeting HCP referral and patient engagement with PR and their theoretically linked behaviour change techniques (BCTs) can help us understand the mechanisms of action underpinning effective interventions, providing useful insight to the evidence base to improve engagement with PR.^33^ Furthermore, exploring theoretical congruence between factors influencing behaviour and current interventions will help identify opportunities for intervention and policy refinement.

The aims of this review are therefore threefold:

1. To conduct a theory-based synthesis of barriers and facilitators to PR referral and engagement behaviours, amongst patients and healthcare professionals through the lens of COM-B and the TDF.
2. To identify and specify the behaviour change techniques in published interventions to improve PR referral and engagement.
3. To triangulate the findings of aims 1 and 2 to understand the extent to which existing interventions use appropriate BCTs to target reported barriers and enablers (theoretical congruence) and to identify further intervention opportunities.

## Methods

**Figure 3:**
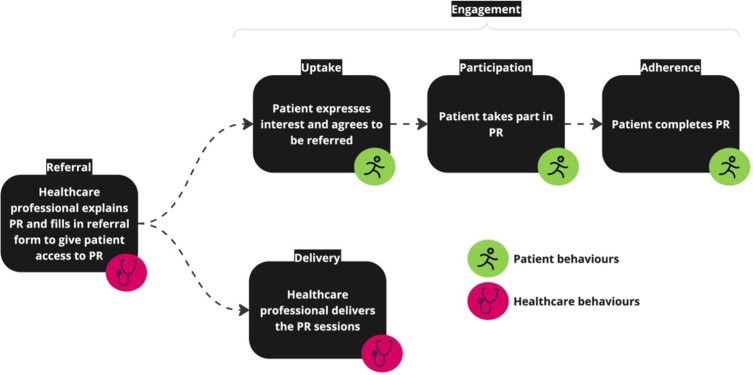
High level behaviours related to referral to and engagement with PR.

**Figure 4:**
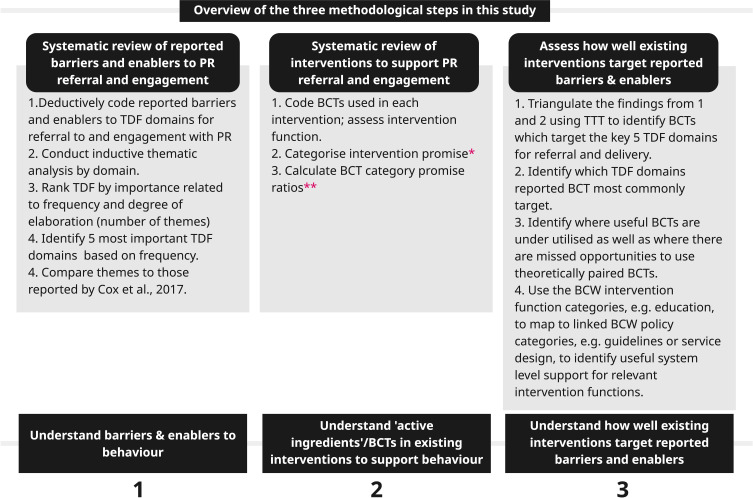
Overview of methods. *Very promising - All PR behaviour outcomes showed significant improvement or behaviour change was greater than for comparator group; Quite promising - At least 1 behaviour outcome showed significant improvement or behaviour change was greater than for the comparator group; Not promising - no change to behaviour outcomes; ** Promise ratios calculated as follows, Number of Very or Quite promising interventions featuring BCTs Cluster/Number of not promising intervention functions featuring BCT cluster.

We conducted two systematic reviews and triangulated the findings to understand how well interventions targeted reported barriers and enablers to behaviour change, from a theoretical perspective. The first review reported literature related to barriers and enablers to PR referral, delivery (Actor – Healthcare professionals) and engagement (Actor – People living with COPD) (PROSPERO Reg CRD42024547289). The relevant behaviours are indicated in Figure 3. The second reported the BCTs used in existing interventions to improve PR referral, delivery and engagement (PROSPERO Reg. CRD42024556812). The triangulated analysis used the TaTT to assess how well the chosen BCTs in each intervention were theoretically matched to the barriers and enablers identified^32^ An overview of the methods is presented in Figure 4. Reporting follows the structure recommended in the PRISMA guidelines.^35^ (Additional information about the models and frameworks used in this review can be found in the Supplementary File Sections 1, Tables S1, S2 and Figures S1–S3).

### Step 1: Systematic Review of Barriers and Facilitators to PR Referral and Engagement

#### Search and Selection

This first review updated Cox et al (2017)^8^ therefore, the search was limited to studies published since July 2016 (the date of the Cox et al last search). The Ovid interface was used to search the databases MEDLINE, EMBASE, and PsycINFO. The search terms used replicated the Cox et al terms, with the addition of a filter for qualitative studies Articles were screened by title/abstract and then full text, using Rayyan software. Ten percent of studies were dual screened to increase reliability (see Supplementary Section 2, Tables S3, S4 and Figure S4).

#### Quality Assessment

The methodological quality of studies was assessed using the Critical Appraisal Skills Programme tool for qualitative studies.^36^ An average quality score was assigned to each study using one of three quality categories: low (10 points), moderate (20 points) and high (30 points) according to reported methodology^37^ (Supplementary File and Table S5).

#### Data Analysis

Data were extracted according to the published protocol. We grouped the analysis into factors influencing HCP referral to PR and patient engagement with PR (including all three of the engagement behaviours) (Figure 3). The behaviour on the PR pathway being explored in each included study was extracted and categorised using the Action, Actor, Context, Target, and Time (AACTT) framework.^38^ Reported barriers/enablers were extracted from published paper in the form of raw data (eg participant quotes) or author interpretations and narrative summaries in the results and discussion sections. Data on barriers/enablers were analysed using a combined deductive framework and inductive thematic analysis approach.^31^,^37^ Barriers and enablers reported in each study were extracted and deductively coded to the 14 TDF domains they were judged to best represent.^39^ An inductive thematic analysis was then carried out within each domain to generate themes representing different influences on PR referral or engagement behaviours.^40^ Final themes were agreed by consensus within the team. Key domains were identified by considering a combination of domain frequency and degree of thematic elaboration (ie number of themes per domain), according to previously reported methodology^8^,^37^ (Expanded analysis detail Section 2, Supplementary File; Coding framework Table S8).

### Step 2: Identifying the Behaviour Change Techniques Used Existing Interventions

#### Search and Selection

The search was based on that of Watson et al 2023^18^ but limited to studies published since their search (August 2021) We also included all the studies identified by Watson et al (2023) for which full text was available. Databases searched were Cochrane Central Register of Controlled Trials through the Cochrane Register of Studies Online (CENTRAL); MEDLINE Ovid; Embase Ovid; CINAHL EBSCO; PEDro (PRISMA flow diagram Figure S5, and inclusion criteria Table S7 and Supplementary File).

**Figure 5:**
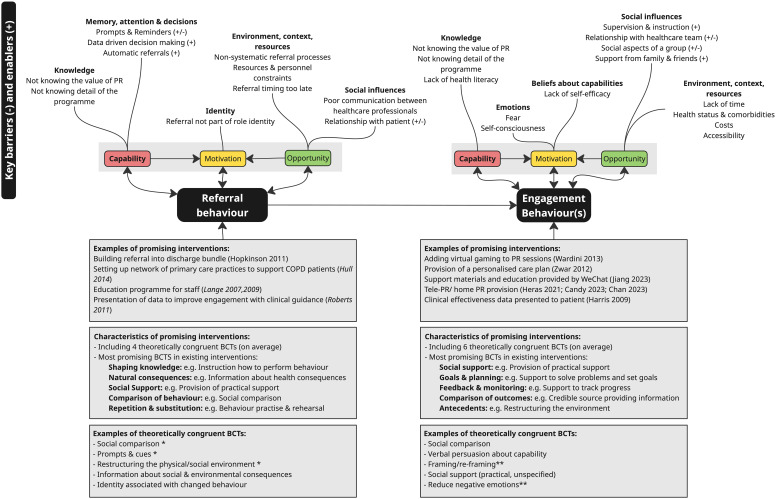
Summary overview of key findings. * target domains currently under-targeted by referral interventions (memory, attention and decision making/Environment, context and resources) therefore represent key opportunities. **target domains currently under-targeted by engagement interventions (emotions) therefore represent key opportunities.

#### Quality Assessment

Risk of bias was assessed for RCTs and quasi-RCTs using the criteria outlined in the Cochrane Handbook for Systematic Reviews for Interventions.^41^ Other non-randomized trials and observational studies were assessed using the ROBINS-I tool^42^ (See Figures S6 and S7).

#### Data Analysis

Intervention descriptions in published reports were read line-by-line and intervention components categorised into BCTs using the Behaviour Change Technique Taxonomy V1 (BCTTv1.)^43^ as well as broader intervention functions using the BCW as coding frameworks.^26^ The PR related behaviours being targeted were identified and categorised using the AACTT framework as per Systematic Review 1.^38^ Interventions were categorised as unpromising, quite promising or very promising, based on whether a significant change in behaviour was reported for one or more outcomes (effect size, confidence intervals and/or p values), using methodology reported by Crayton et al.^44^ Promise ratios (PRatio) were calculated based on previously reported methodology.^43–46^ (See Supplementary File for formula, Section 2.2.5). The PRatio gives an indication of effectiveness of a BCT by calculating the number of promising interventions a BCT is identified in compared to the number of unpromising interventions a BCT is identified in. In our calculations, we have used the BCTTv1.0 BCT grouping by type for the analysis, rather than the individual BCTs, for example Group 4 is the BCTs which shape knowledge such as *Instruction how to perform behaviour* and *Information about antecedents*. This allowed us to calculate PRatios for more of the BCTs which were not commonly used. Meta regression was not possible due to outcome heterogeneity.

### Step 3: Investigating Theoretical Congruence

Theoretical congruence was explored by using the TaTT to identify BCTs which target the key TDF domains identified in systematic review 1 and triangulating this information with the BCTs identified in interventions included in systematic review 2.^32^

#### Data Analysis

In a triangulated analysis, the TaTT was used to explore how likely BCTs in existing interventions were to target the TDF domains (Mechanisms of Action) identified as key for PR referral and engagement behaviours. Each identified BCT was classified as having high (targets 2 or more key TDF domains 1), medium (targets at least 1 key TDF domain) or low theoretical congruence (targets none of the key TDF domains) based on the TDF domains identified as key in step 1.^47^ We also reviewed which BCTs had evidence of effectiveness for the key TDF domains highlighted, as suggested by the TaTT, for each behaviour but which were not used in existing interventions. These BCTs represented missed opportunities and may offer potential for consideration in future interventions. The BCW methodology also supports the intervention designer to identify appropriate policy categories (broader system level strategies) based on the behavioural analysis.^26^ We used this approach to identify potential system level strategies that could support intervention delivery to support PR referral and engagement. Further analysis detail is available in Section 2.3.1 and Supplementary File.

## Results

An overall narrative summary of the findings is given in Figure 5.

**Table 1:** Included Studies in Step 1: Review of Barriers and Enablers to Referral and Uptake and Engagement Behaviour

Author/Year	Country	Study Design	Method	Sample	Sample Size	PR Behaviour Focus	Actor
Barradell 2022^48^	US	Qualitative	Semi-structured interviews	HCPs, Patients	25	Referral and uptake	HCPs and Patients
Bradley 2023^49^	UK	Qualitative	Semi-structured interviews	Patients	9	Uptake	Patients
Brighton 2020^50^	UK	Qualitative	Semi-structured interviews	Patients	19	Participation	Patients
Cochrane 2016^51^	Australia	Mixed methods	Structured & semi-structured interviews	HCPs, Patients	7	Participation	HCPs and Patients
Early 2020^52^	UK	Mixed methods	Survey, semi-structured interviews, focus groups	HCPs, PR Commissioners, Patients	80	Referral and uptake	HCPs and Patients
Gabriel and Finkelstein 2024)^53^	US	Qualitative	Semi-structured interviews	HCPs	11	Referral	HCPs
Harb 2017^54^	Australia	Qualitative	Semi-structured interviews	Patients	26	Participation	Patients
Issac 2021^55^	Australia	Qualitative	Semi-structured interviews	HCPs (incl. a social worker)	8	Referral	HCPs
Janaudis-Ferreira 2019^56^	Canada	Qualitative	Semi-structured interviews	HCPs, Patients	24	Participation	HCPs and Patients
Jiang 2021^57^	China	Qualitative	Semi-structured interviews	HCPs, Patients	51	Participation	Patients
Krag 2023^58^	Denmark	Qualitative	Semi-structured interviews	Patients	11	Not specified	Patients in PR
Lahham 2020^59^	Australia	Qualitative	Semi-structured interviews	HCPs	30	Referral	HCPs (mixed)
Leemans 2023^60^	Belgium	Qualitative	Semi-structured interviews	HCPs	30	Referral	HCPs
Liu 2021^61^	UK	Mixed methods	Survey, Semi-structured interviews, focus group	HCPs, Patients	19	Referral & uptake	HCPs (multi-disciplinary) and patients
Machado 2022^62^	Portugal	Qualitative	Semi-structured interviews	Patients	11	Not specified	Patients
Maglakelidze 2022^63^	Georgia	Mixed methods	Semi-structured interviews	Patients	9	Participation	Patients
Mathar 2017^64^	Denmark	Qualitative	Semi-structured interviews	Patients	19	Uptake	Patients
Molin 2020^65^	Denmark	Qualitative	Semi-structured interviews	Patients	14	Not specified	Patients (with moderate COPD)
Molin 2016^66^	Denmark	Qualitative	Semi-structured interviews	HCPs	8	Not specified	HCPs (GPs)
O’Connor 2019^67^	UK	Mixed methods	Semi-structured interviews	Patients	11	Uptake	Patients
Ogunbayo 2017^68^	UK	Qualitative	Semi-structured interviews	HCPs	20	Not specified	Not specified
Oliveira 2021^69^	Canada	Qualitative	Semi-structured interviews	HCPs, Policymakers, Patients	16	Referral & uptake	HCPs, patients (with recent exacerbation)
Padhye 2023^70^	India	Qualitative	Semi-structured interviews	HCPs, Patients	29	Not specified	Not specified
Pagano 2023^71^	Australia	Qualitative	Semi-structured interviews	HCPs, Patients	19	Not specified	Not specified
Poureslami 2017^72^	Canada	Qualitative	Focus groups and interviews	Patients	28	Participation	Patients
Simonÿ 2019^73^	Denmark	Qualitative	Ethnography & narrative interviews	Patients	21	Participation	Patients
Simonÿ 2020^74^	Denmark	Qualitative	Ethnography & semi-structured interviews	Patients	21	Participation	Patients
Simonÿ 2022^75^	Denmark	Qualitative	Mixed methods	Patients	21	Participation	Patients
Singh 2023^76^	India	Qualitative	Semi-structured interviews	HCPs, Patients	23	Referral & uptake	HCPs, Patients
Spitzer 2020^77^	US	Qualitative	Semi-structured interviews	Patients	15	Uptake & participation	Patients
Spitzer 2023^78^	US	Qualitative		HCPs	38	Referral and participation	HCPs and Patients
Stokes 2019^79^	N.Zealand	Qualitative	Semi-structured interviews	HCPs, Patients	36	Uptake	HCPs and Patients
Watson 2020^5^	UK	Qualitative	Semi-structured interviews	HCPs	19	Referral	HCPs (Primary care HCPs)
Watson 2022^80^	UK	Mixed methods	Semi-structured interviews and survey	HCPs	252	Referral	HCPs (Primary care HCPs)

### Step 1: Barriers and Facilitators to PR Referral and Engagement

We identified 34 studies for inclusion (Table 1). Results of the selection process are presented in the PRISMA flow chart in the Supplementary File (Figure S4) together with further data about the included studies including the quality assessment (Table S5), and AACTT data (Table S9).

#### Study Characteristics

Studies were from the United Kingdom (24%), Denmark (21%), Australia (15%) and the United States (12%). Over 40% of studies considered influences on engagement behaviour, 44% included influences on engagement and referral behaviour, and 27% considered only referral behaviour. No studies exclusively examined delivery of PR, but some data related to delivery was included in mixed studies.

#### Quality

The median score was 27 (range 25–35), representing overall high quality (Table S5).

**Table 2:** Ranking of TDF Domain According to Frequency of Identification Amongst Studies & Thematic Elaboration. First 5 Domains in Bold Represent 60% of Themes Generated and are Classified as “Key” for Step 3

Rank	Referral (18 Studies)					Engagement (31 Studies)				
		No. of studies	Barriers	Enablers	Elaboration	TDF (COM-B)	No. of Studies	Barriers	Enablers	Elaboration
1.	**Environmental context and resources (Phys.O)**	17	44	25	6	**Environmental context and resources (Phys.O)**	27	76	29	10
2.	**Knowledge (Psy.C)**	11	16	7	3	**Social influences (Soc.O)**	27	13	59	6
3.	**Social influences (Soc.O)**	11	7	14	2	**Emotions (Aut.M)**	17	16	6	4
4.	**Social/Professional role and identity (Ref.M)**	9	13	2	1	**Knowledge (Psy.C)**	16	23	7	4
5.	**Memory, attention and decision proc. (Psy.C)**	8	1	14	4	**Beliefs about capabilities (Ref.M)**	15	26	2	1
6.	Skills (Psy.C/Phy.C)	5	2	4	2	Skills (Psy.C/Phy.C)	12	3	10	2
7.	Beliefs about consequences (Ref.M)	4	2	4	1	Beliefs about consequences (Ref.M)	11	6	7	1
8.	Beliefs about capabilities (Ref.M)	4	4	1	1	Optimism (Ref.M)	9	7	3	1
9.	Reinforcement (Aut.M)	4	2	6	2	Memory, attention and decision processes (Psy.C)	8	6	4	5
10.	Goals (Ref.M)	3	1	2	1	Reinforcement (Aut.M)	8	5	6	1
11.	Intention (Ref.M)	3	1	3	1	Goals (Ref.M)	6	0	8	1
12.	Emotions (Aut.M)	1	1	0	1	Intention (Ref.M)	6	5	2	1
13	Optimism (Ref.M)	1	1	0	1	Social/Professional role and identity (Ref.M)	5	5	0	1
14.	Behavioural regulation (Psy.C)	1	1	0	1	Behavioural regulation (Psy.C)	3	0	4	1

**Abbreviations**: COM-B domains – Psy.C, psychological capability; Phy.C, physical capability; Ref.M, reflective motivation; Aut.M, automatic motivation; Soc.O, social opportunity; Phys.O, physical opportunity.

#### Findings

##### Referral

Ranking of the importance of the TDF domains based upon frequency from the deductive coding is shown for referral in Table 2 (Table S10, shows an audit trail of the coding across each study). The domains are presented in rank order, according to the number of papers reporting a barrier/enabler within that domain, along with supporting quotes. The domain *Environmental context and resources* (eg lack of standardised referral procedures) was most frequently identified, appearing in 94% of included studies, followed by *Knowledge* (61%) (eg not Knowing the impact of PR), and *Social influences* (61%) (eg lack of interprofessional communication). The themes from the inductive thematic analysis for the top 5 domains are listed in Table 3. These themes and those from less frequently cited domains are further described in the Supplementary File (Tables S11 and S12). In terms of elaboration, the first five domains by frequency account for 60% of inductive themes generated, indicating good convergence between domain frequency and level of elaboration. Domain ranking by elaboration therefore remains consistent with ranking by frequency.

**Table 3:** Inductive Themes by TDF Domain for Referral

Rank	TDF	Themes (Number of Studies)	Description	Example
1.	Environmental context and resources	Resources and personnel (mixed) (13)	Lack of staff, resources, promotional materials. Lack of available PR courses	“*If the resources were out there, then I would definitely refer to PR*”*53*
		[Non] Systematic referral (barrier) (10)	Lack of standardised referral processes	“*We lack a system that is organized*”*66*
		Timing of referral discussion (mixed) (7)	Timing of referral within COPD timeline influences referral success	“*Always been our barrier that we’re approaching people that are feeling breathless and terrible and they do not want to think about anything*”*48*
2.	Knowledge	[Not] Knowing the value of PR for health outcomes (barrier) (8)	HCPs may lack detailed knowledge about PR and its value	“*I think that PR has no more effect than saying, for example, go outside for some cycling*”*60*
		Knowledge of programmes, adjunct services, and referral process (barrier) (4)	Pulmonary specialists were often not aware of existing programmes and how to refer	“*one of the big barriers is not knowing how to refer*”^53^
3.	Social influences	Interprofessional communication (mixed) (8)	HCPs attributed poor knowledge of PR to lack of interprofessional communication. Good communication facilitated referral.	“*I wouldn’t know how many people we refer, is that referral going up, Nobodies giving us feedback from the rehab team*”*80*
		HCP-Patient relationship (mixed) (5)	Positive communication between HCP and patient can facilitate referral and lack of continuity of care is a barrier.	“*Increasingly, continuity of care is broken, so you see a patient who you’re meeting for the first or second time and the conversation is very different to a patient you have known the last 10 or 15 years*”*52*
4.	Social/Professional role and identity	Referral [not] part of professional role (barrier) (9)	Some HCPs believe PR is not a medical programme, not their responsibility and others better placed to refer.	“*I mean, either social work or psych [could help] with that*”*56*
5.	Memory, attention and decision processes	Prompts (enabler) (6)	Prompts eg pop-ups on screen or patient care templates reminded HCPs to refer, as often it was forgotten.	“*I don’t want to tick boxes but I do need a prompt*”*80*
		Data-driven decision making (enabler) (2)	Using hospital data and sharing data could inform decision making	“*In many hospitals, clinicians reported using data to inform decisions about PR (Author summary)*”*77*
		Automatic referrals (enabler) (2)	Automatic referrals, such as when integrated into systems reduced cognitive demand on HCP. An opt out system was positively viewed.	“*with the rehab so we’ve introduced to say it’s an opt out*”^48^

##### Engagement

Ranking of the importance of the TDF domains based upon frequency of the deductive coding is shown for engagement in Table 2. The most frequently identified TDF domains for engagement were *Environmental context and resources* (eg poor accessibility of venue or transport challenges), *Social influences* (eg level of trust in relationship with HCP) and *Emotions* (eg fear about being able to exercise safely). The themes for the top 5 domains are listed in Table 4 and are further elaborated in the Supplementary File (Tables S13 and S14). The first five domains by frequency account for 64% of inductive themes generated, indicating good convergence between domain frequency and level of elaboration.

**Table 4:** Inductive Themes by TDF Domain for Engagement

Rank	TDF (COM-B)	Themes (Number of Studies)	Description	Example
1.	Environmental context and resources	Health status, comorbidities, and immobility (barrier) (13)	Low mood and multiple comorbidities, including immobility was a common barrier.	“*Maybe I can do it another time when I get a better mood and health*”*64*
		Time (barrier) (10)	Time commitment in context of other more pressing priorities. Challenge of combining with work.	“*When should I attend it? I have no time for it*”*64*
		Accessibility (barrier) (16)	Poor accessibility to the venue and associated lack of transport	“*If I’m very ill and I do not have transportation and I cannot come by public transportation*”*56*
		Resources and personnel (mixed) (12)	Costs associated with attending eg parking and lack of specialist staff eg interpreters were barriers if absent and enablers if present.	“*Cost factor is very important*”*76*
		Tailored and flexible programme (mixed) (10)	The tailored exercise programme alleviated anxiety improving confidence but lack of flexibility in timing of sessions was a barrier.	“*I want to have the freedom to do it when it suits me*”*64*
2.	Social influences	Supervision and instruction (enabler) (10)	The accessibility to knowledgeable staff and safe supervision by qualified professionals was a facilitator	“*It gives me an opportunity to exercise 3 times a week under professional supervision*”*72*
		HCP-patient relationship (mixed) (14)	When relationships between the HCP and patient is positive and trusting it supports referral but lack of trust is a barrier.	“*I love my pulmonologist. I would stand on my head for him*”*77*
		Social aspect of group (enabler/mixed) (14)	The group setting was largely facilitating, although some were anxious about exercising in front of others.	“*You meet the people, and they have the same problems, so you get to talk about the same problems*”*77*
		Support from family/friends (enabler/mixed) (6)	Social support outside the PR class was facilitating as friends and family supported with transportation and encouragement. No support hindered attendance.	“*I looked at the letter with my son and he said* “Go for it Mam,’ *so I filled it in and he posted it in the letterbox*”*69*
3.	Emotions	Fear (barrier) (15)	Patients were fearful of exercising as well as being pressurised to stop smoking, exercising in a group setting.	“*I have anxiety about not being able to breathe, or to get suffocated*”*58*
		Enjoyment (enabler) (5)	PR classes brought participants joy and laughter and fun.	“*It was funny, enjoyable and also a bit serious because we are going there with a serious intent. However, it makes a difference that it becomes a bit of fun*”*74*
		Self-consciousness (barrier) (2)	Exercising in front of others and being out performed by older participants reduced motivation to attend.	*Two women noted that they felt self-conscious exercising in a group setting (author summary)**77*
4.	Knowledge	[Not] Knowing the value of PR for health outcomes (barrier) (15)	Knowing clearly the benefits of PR facilitated attendance but patients reported not understanding the value and not noting what would happen at PR.	“*Well, one, I don*”*t think I need it, because I do my own exercise*”*56*
		[Lack of] health literacy (barrier) (8)	Poor health literacy and little understanding of COPD and its management were barriers but when patients were knowledgeable and motivated it supported PR attendance.	“*With COPD there is not much that can make it better*”*64*
		Knowledge of programmes and adjunct services (barrier) (4)	Lack of awareness of the existence of PR or related services eg transport, acted as a barrier to participation.	*Interviewer: And are you familiar with the idea of pulmonary rehabilitation? Interviewee: No, I have no idea what it is. No*.*77*
5.	Beliefs about capabilities	Self-efficacy (mixed) (16)	Patients often believed they were too breathless to exercise or to get to PR. High levels of self-efficacy were less common though facilitative.	“*I’m not strong enough. I cannot, I even have a hard time to get to the door*”^56^

### Step 2: Intervention Characteristics

Twelve papers met the inclusion criteria. Seventeen studies from Watson et al (2023), where the full text was available, were also included, resulting in a total of 29 studies meeting inclusion criteria (Table 5) (See Supplementary File for PRISMA flow chart, Figure S5.

**Table 5:** Included Studies in Step 2: Review of Interventions to Support Referral, Uptake/Participation and Adherence

Author/Year	Country	Study Design	Sample Group	Intervention Duration	PR Behaviour Focus	Comparison	Outcome
Aboumatar 2021^81^	USA	RCT	Patients	9 months	Uptake and Adherence	No Peer support	PR adherence
Angus 2012^82^	UK	Feasibility Study	Nurses	Baseline	Referral	No Control	Referral rate
Barker 2020^83^	UK	RCT	Patients	90-day	Referral, Uptake and Adherence	Usual Care	Referral rate, PR adherence
Belloumi 2023^84^	Tunisia	Feasibility Study	Patients	N/A	Uptake	No Control	Parameters among adherent patients (RA) versus non-adherent (RNA) patients
Candy 2023^85^	N Zealand	Non-randomised trial	Patients	8 weeks	Uptake and Adherence		PR adherence and attendance
Chan 2023^86^	Malaysia	Feasibility study	Patients	8 weeks	Uptake and Adherence	No Control	PR adherence and attendance
Colombo 2023^87^	Italy	Pilot study	Patients	N/A	Uptake	No Control	PR adherence
Foster 2016^88^	UK	Mixed method	GPs and PNs	N/A	Referral and Uptake	No Control	Referral rate and acceptance
Graves 2010^89^	UK	Intervention study	Patients	N/A	Uptake and Adherence	Not recorded	PR adherence and attendance
Harris 2009^90^	Australia	Controlled Clinical Prospective trial	Patients	12 months	Referral and Uptake	Usual Care	Referral rate and acceptance
Heras 2021^91^	Denmark	RCT	Patients	8 months	Adherence	Standardized PR program	PR adherence
Hopkinson 2011^92^	UK	Implementation study	GPs	1 year	Referral	No Control	Referral rate
Hull 2014^93^	UK	Longitudinal Audit	Patients	3 years	Referral	No Control	Referral rate
Jiang 2023^94^	China	RCT	Patient	3 months	Adherence	PR without PDA	PR adherence
Kaasgaard 2021^95^	Denmark	RCT	Patients	2 weeks	Uptake and Adherence	Control arm	PR adherence
Lange 2007^23^	Denmark	Cross-sectional	GPs and Patients	1 year	Referral	Usual care	Referral rate
Lange 2009^96^	Denmark	Cross-sectional	Outpatient Clinics	1 year	Referral	Usual care	Referral rate
Maglakelidze 2022^63^	Georgia	RCT	Patients	6 months	Uptake and Adherence	Usual care	Follow-up rate at 6 months, PR adherence
Pagano 2023^71^	Australia	Pilot study	GP practices	3 months	Referral and Uptake	No Control	Referral rate, PR attendance
Pena 2023^97^	Colombia	Cross-sectional	Patients	N/A	Uptake	No Control	PR adherence
Polo 2023^98^	USA	RCT	Patients	6 months	Uptake and Adherence	SPR	PR adherence and attendance
Ringbaek 2016^99^	Denmark	RCT	Patients	7-week	Adherence	Standard PR	PR adherence
Roberts 2015^100^	UK	Quasi-experimental	GP practices	3 months	Referral	Usual Care	Data on pulmonary rehabilitation
Sewell 2017^101^	UK	Feasibility Study	Patients	1 year	Referral	No Control	Referral rate
Sonnick 2021^102^	USA	QI study	Health Care Providers	N/A	Referral	Historic Control	Referral rate
Ulrik 2010^103^	Denmark	Intervention study	GPs	N/A	Referral	No Control	Referral rate
Wardini 2013^104^	Canada	Intervention study	Patients	3-4 week	Uptake and Adherence	Standard PR	PR adherence and attendance
Zwar 2012^105^	Australia	Cluster RCT	GPs	12 months	Uptake	Usual care	Attendance at PR
Zwar 2016^106^	Australia	Cluster RCT	GPs and PNs	Baseline only	Uptake	Case-finding training for nurses	Attendance at PR

**Abbreviations**: QI, quality improvement; RCT, randomised controlled trial.

#### Study Characteristics

The United Kingdom (n = 7), and Denmark (n = 6) were the countries with the largest number of studies. Studies were also conducted in Canada, USA, Australia, Malaysia, Colombia, Italy, New Zealand, Tunisia, Georgia, and China. Across the 29 studies, patients and general practitioners (GPs) were the most common sample groups. Across the studies, 62% of interventions were targeted at people living with COPD (n = 18) and 38% at healthcare professionals. The majority took place in either GP practices (n = 7) or in-person clinics (n = 7). Nine studies focused on referral behaviour, 16 on engagement behaviours (3 on adherence only, 5 on uptake only and 8 on uptake and adherence) and 4 on both referral and engagement (Table 5). Of the interventions considering patient engagement, none considered intervention components that were intended to improve support from family and carers. The AACTT coding is presented in Table S15, in the Supplementary File.

#### Quality

Only one of the RCTs yielded a high risk of bias.^99^ Of the nineteen non-randomised studies that were assessed using the ROBINS-I tool, eight (42%) were assessed as having a moderate risk, seven (37%) as having a severe risk and four (21.1%) as having a critical risk. No studies were excluded based on quality (Supplementary File, Figures S6 and S7).

**Table 6:** Intervention Descriptions

Author	Promise	Intervention Target	Intervention Description	Intervention Functions	BCT Examples
Aboumatar, 2021^81^	Quite	Engagement	Peer support	Enable	Problem solving, Social support, Credible source
Angus, 2012^82^	Quite	Referral	Computer guided consultation in general practice	Enable	Social support, Prompts & cues, Monitoring behaviour
Barker, 2020^83^	Not	Referral & engagement	Video intervention post-hospitalisation	Educate	Information about health consequences, Credible Source
Belloumi, 2023^84^	Quite	Engagement	Home-designed PR service	Enable	Social support, Instruction on how to perform behaviour, Self-monitoring
Candy, 2023^85^	Quite	Engagement	Tele-health PR programme	Enable	Social support, Instruction on how to perform behaviour
Chan, 2023^86^	Quite	Engagement	Home based PR	Enable	Goal-setting, Action planning, Demonstration of behaviour
Colombo, 2023^87^	Not	Engagement	Virtual reality training in PR	Enable	Goal setting, Biofeedback, Social support
Foster, 2016^88^	Quite	Referral & engagement	Audits and co-designed strategies to increase referral	Educate	Social support, Instruction of how to perform behaviour, Prompts & cues
Graves, 2010^89^	Quite	Engagement	Group opt-in, Pre-PR	Educate	Social support, Information about health consequences, Credible sour.
Harris, 2009^90^	Quite	Referral & engagement	Providing COPD patients with reviews of evidence	Persuade	Information about health consequences, Prompts & cues
Heras, 2021^91^	Quite	Engagement	Tele-PR programme	Enable	Action planning, Biofeedback, Demonstration of behaviour
Hopkinson, 2011^92^	Very	Referral	Discharge care bundle	Env. Restructure	Monitoring of behaviour by others without feedback, Instruction
Hull, 2014^93^	Very	Referral	Developing GP networks to support COPD management	Env. Restructure	Adding objects to the environment, Social support (unspecified)
Jiang, 2023^94^	Very	Engagement	PR decision making aid for PR, Web resources sent via WeChat	Educate	Instruction, Information about health consequences
Kaasgaard, 2021^95^	Not	Engagement	Use of singing as part of the PR training programme	Enable	Social support (unspecified), Social support (practical)
Lange, 2007^23^	Very	Referral	Education for GPs	Educate	Behavioural practice/rehearsal, Credible source, Social support
Lange, 2009^96^	Very	Referral	Education for healthcare teams	Educate	Social support, Instruction on how to perform behaviour
Maglakelidze, 2022^63^	Quite	Engagement	Culturally tailored PR programme (in Georgia)	Enable	Monitoring of behaviour by others without feedback, Credible source
Pagano, 2023^71^	Not	Referral & engagement	GP-Physiotherapist partnership to support patient	Educate, Env. Restructure	Action planning, Social support, Behaviour practice/rehearsal
Pena, 2023^97^	Quite	Engagement	Monitoring of patient adherence rates	Enable	Social support, Monitoring of outcomes by others
Polo, 2023^98^	Quite	Engagement	Tele-PR	Enable	Social support, Adding objects to the environment, Rewarding completion
Ringbaek, 2016^99^	Not	Engagement	Provision of tablet computers to PR participants	Enable, Env. Restructure	Problem solving, Advise to change behaviour, Social reward
Roberts, 2015^100^	Very	Referral	Patient score card	Env. Restructure	Feedback on outcomes (behaviour), Instruction on how to perform beh.
Sewell, 2017^101^	Very	Referral	Discharge care bundles	Env. Restructure, Enable	Advise to change behaviour, Social support (unspecified)
Sonnick, 2021^102^	Very	Referral	Real-time teaching intervention for healthcare teams	Educate	Salience of consequences, Credible source, Instruction.
Ulrik, 2010^103^	Not	Referral	Education programme for GPs	Educate	Demonstration of the behaviour, Credible source, Social support
Wardini, 2013^104^	Very	Engagement	Virtual gaming as part of PR	Enable	Instruction on how to perform behaviour, Credible source
Zwar, 2012^105^	Very	Engagement	Nurse with clinical COPD training working in partnership with GP	Env. Restructure	Behaviour practice/rehearsal, Social support (unspecified)
Zwar, 2016^106^	Not	Engagement	Disease management plan for newly diagnosed; Computer tablet	Env. Restructure	Prompts & cues, Credible source, Information about health consequences.

**Notes**: Education – increasing knowledge or understanding; Persuasion – using communication to induce positive or negative feelings or stimulate action; Environmental (Env). restructuring – changing the physical or social context; Enablement – Increasing means or reducing barriers to increase capability (beyond education and training) or opportunity (beyond environmental restructuring.

#### Findings

##### Interventions

A summary of the interventions is provided in Table 6. Interventions were categorised as using the functions (means to change behaviour) as detailed in the BCW^26^. Functions were *Education (*eg education sessions for GPs^23^), *Enablement* (eg cultural tailoring of the programme^63^), *Environmental restructuring* (eg provision of a computer tablet to PR participants^99^) and *Persuasion* (eg presentation of PR effectiveness evidence^90^). In terms of BCW policy categories, interventions focused mostly on *Service provision*, with two of these also focussing on *Environment/social planning* by providing group networking support to patients or GP practices.^92^,^93^

##### Intervention Effectiveness

**Figure 6:**
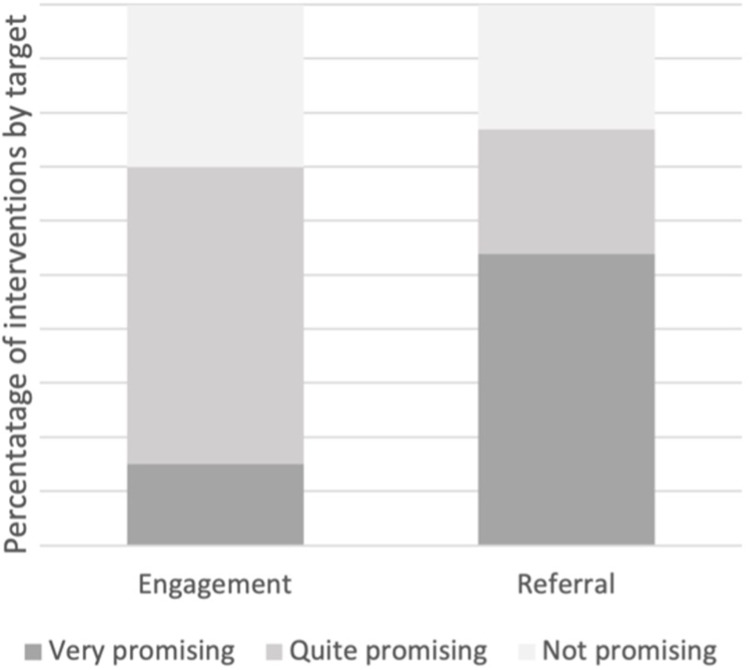
Summary of promise of interventions across intervention types.

Interventions were rated as “very promising”, “quite promising” and “not promising” (Figure 6 and Table 6). Fifty four percent of interventions targeting referral were “very promising”. “Very promising” interventions included those offering group education sessions for staff,^23^,^96^ developing a “discharge bundle” to include automatic PR referral,^92^ and real-time teaching embedded into the patient encounter.^102^ By comparison to 15% of interventions targeting engagement were considered “very promising”; these included providing support materials to patients directly via smart phone^94^ and presenting clinical data imaginatively to improve patient engagement.^100^

##### BCT Selection and Frequency

Similar BCTs were commonly used in interventions targeting referral and those targeting engagement, with the most frequently used being the provision of social support, giving detailed instruction about how to perform the behaviour, and providing practical demonstration. The most frequently used BCTs for interventions targeting referral and engagement are shown in Table 7, together with examples of their use. A full list of all the BCTs coded for each included intervention is provided in the Supplementary File (Tables S16 and S17).

**Table 7:** Most Frequently Used BCTs and Examples of How These Were Used in Interventions

BCT	Referral (13 Interventions) *		Engagement (20 Interventions) *	
	Freq	Examples of Use	Freq	Examples of Use
Social support (unspecified)	9	Meeting with staff in participating GP practices where guidelines were discussed by a pulmonary specialist^23^	13	1-2-1 and group conversations with peer support worker^81^
Instruction how to perform the behaviour	9	Giving healthcare professionals skills training and specific instructions^100^	13	Physio giving guidance of how to do the exercises^104^
Demonstration of the behaviour	7	Symposium of lectures and workshops with practical sessions like interpreting spirometry^23^	11	Giving participants demonstrations of how to do all the exercises^91^
Information about health consequences	5	Manual summarised evidence about COPD treatments^90^	8	General pre-PR education sessions offered to patients^103^
Monitoring of the behaviour by others without feedback	4	Management recommendations were recorded^82^	7	Peer supporter monitored participants comfort with the activities^81^
Behaviour practise/rehearsal	6	Practical workshops carried out with HCPs^103^	9	Video recordings provided to allow practise between sessions^99^
Credible sources	5	Steering committee member presents different aspects of the guidelines at group meeting^23^	6	Introductory video by trusted health professional (respiratory nurse)^94^

**Notes**: *4 interventions targeting both referral and engagement.

##### BCTs Used in Effective Interventions

The most promising BCTs in interventions targeting referral included those from Group 4 in the BCT Taxonomy, relating to shaping Knowledge such as *Instruction on how to perform the behaviour* and *Information on antecedents* and Group 5 related to natural consequences such as *Information about health consequences* and *Salience of consequences*. Lesser effective BCTs were from Group 1 – goals and planning and Group 9 – comparison of outcomes. For interventions targeting engagement, the most promising BCTs were from Group 3 – social support, such as *Social support (unspecified)* and *Social support (emotional)* (Table 8).

**Table 8:** Promise Ratios Indicating Likely Promise of BCT Groups in Interventions to Increase Referral and Engagement

Referral		Engagement	
BCT Group	Promise Ratio	BCT Group	Promise Ratio
4. Shaping knowledge	4.0	3. Social support	3.1
5. Natural consequences	4.0	1. Goals & Planning	3.0
3. Social support	3.3	2. Feedback & monitoring	3.0
6. Comparison of behaviour	3.0	9. Comparison of outcomes	3.0
8. Repetition & substitution	1.5	12. Antecedents	3.0

### Step 3: Triangulating Findings to Assess Theoretical Congruence

The TaTT maps link between BCTs and the TDF domains (or mechanisms of action) based on available evidence. A BCT used in an intervention is determined to have theoretical congruence when there is evidence for linkage to a TDF domain identified as key for the behaviour in question. Theoretically, congruent BCTs are more likely to be effective.

In this analysis, BCTs were determined to be theoretically congruent and therefore more likely to support referral or engagement behaviours, when there was evidence for linkage to TDF domains identified as key in the first review – in the top 5 for frequency.

#### Referral

The most promising referral interventions incorporated a mean of 4 theoretically congruent BCTs compared to 2 in less promising interventions, suggesting that the choice of BCTs indicated to be theoretically congruent by the TaTT, may improve effectiveness of the intervention (Tables S18 and S19, Supplementary File).

**Figure 7:**
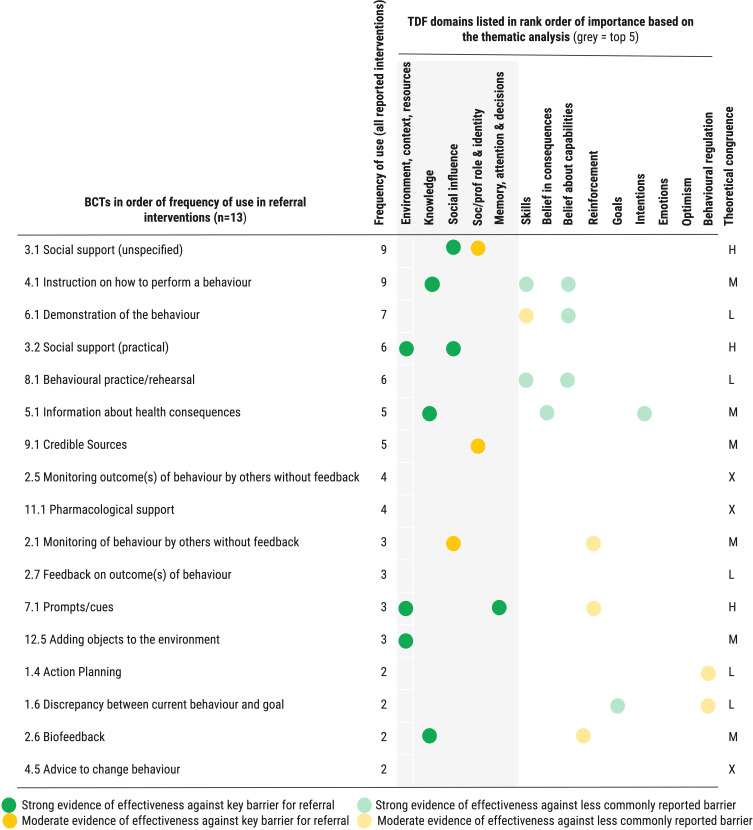
Schematic illustrating choice of BCTs in existing referral interventions with evidence of effectiveness against key domains (barriers) reported. H = BCT congruent with 2 or more key domains; M BCT congruent with at least 1 key domain; L = BCT not congruent with any key domain reported but congruent with other domains. X = BCT not congruent with any domain reported.

The triangulation of review 1 and 2 findings identified several areas where existing interventions are adequately targeting key influences on PR behaviours. For example, social influences such as a lack of professional intercommunication (TDF: social influences) and feelings that PR referral was not part of an individual’s professional role (TDF: social/professional role and identity) were identified as key influences on referral behaviour in review 1. The TaTT identifies that BCTs offering social support can influence both these domains. The provision of social support for HCPs was used in multiple interventions (BCT: *Social support unspecified, Social support practical*) through components such as respiratory nurse teams offering guidance and support to ward teams,^101^ real-time teaching support given to discharge staff^102^ and development of GP network discussion meetings.^93^ Nine interventions targeting referral also included components delivering BCTs shown to influence knowledge, for example staff completing an online module about COPD^106^ and staff taking part in educational sessions about PR^103^ (BCT: *Information about health consequences*) and staff taking part in a real-time ward based teaching intervention offered upon discharge in the ward.^102^ More rarely, other influences such as the referring practitioner forgetting to do the referral (TDF: Memory, attention and decision making) and not having sufficient time within the consultation (TDF: Environment, context and resources) were targeted in existing interventions, by components such as the provision on referral prompts on COPD review templates,^88^ or embedding algorithms into the GP systems^82^ (BCT: *Prompts & cues*). BCTs commonly used in referral interventions which appropriately target the barriers and enablers identified for referral in step 1 (ie which are theoretically congruent) are illustrated in Figure 7. Tables S20 and S21 in the Supplementary File provide detail of recommended BCTs for all the domains identified in review 1 and provide extended detail on pairings of BCTs used in existing interventions. Figure S8 is a schematic indicating overall how well existing interventions target key barriers/domains identified (Supplementary File).

**Figure 8:**
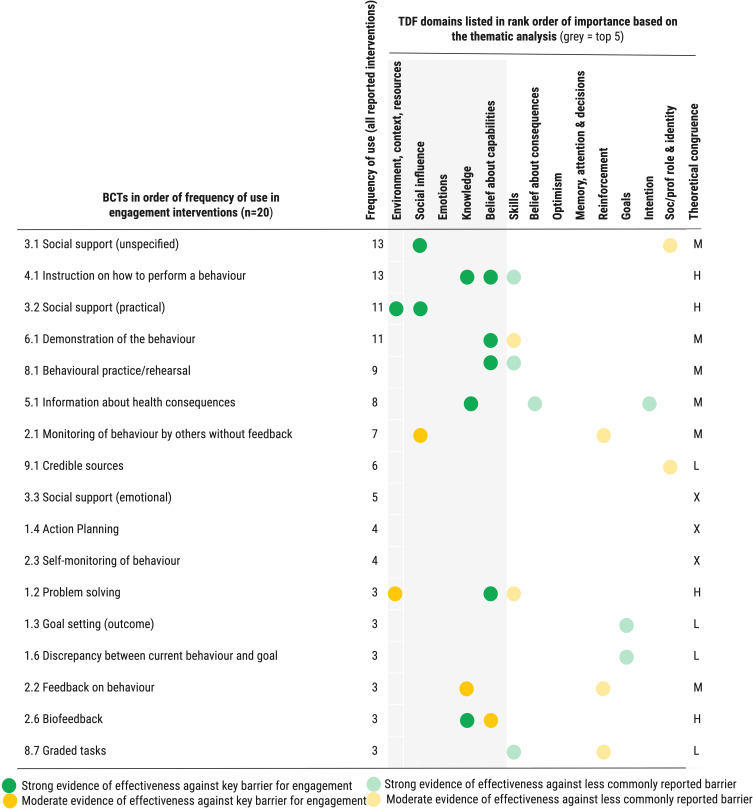
Schematic illustrating choice of BCTs in existing engagement interventions with evidence of effectiveness against key domains (barriers) reported. H = BCT congruent with 2 or more key domains; M BCT congruent with at least 1 key domain; L = BCT not congruent with any key domain reported but congruent with other domains. X = BCT not congruent with any domain reported.

There were minimal mismatches in the case of referral interventions – interventions including components delivering BCTs which did not map to the key influences on referral identified in review 1. One example of this mismatch is including practical workshops to rehearse referral^103^ (BCT: *Behaviour practise and rehearsal*) which would be useful when addressing influences associated with self-belief about performing the behaviour (TDF: Belief in capabilities). The TaTT analysis suggests such an approach is unlikely to influence referral behaviour as lack of self-belief in referral behaviour was not identified as a key influence on referral behaviour.

There were 9 BCTs that were identified as theoretically congruent with key influences on referral behaviour, according to TaTT evidence but which were not used in existing interventions. These BCTs, supported by evidence of effectiveness, are likely to positively influence referral behaviour through the key TDF domains/MoAs identified in review 1. They represent missed opportunities in current interventions (see Table S22 and Supplementary File). Suggestions for how these BCTs may be operationalised in future interventions are shown in Table 9. For example, comparing referral figures across referrer and GP practices (BCT: *Social comparison*) may influence barriers associated with a lack of belief that PR referral is part of the HCP role (TDF: Social/professional role and identity). Table S22 details.

**Table 9:** Opportunities for Choosing Other Theoretically Matched BCTs in Referral Interventions

BCT	Target Domain	Example of Potential Operationalisation
6.2 Social comparison	Social professional role/identity and Social influences	Comparing referral figures with peers within the practice/hospital and across sites
13.5 Identity associated with changed behaviour	Social professional role/identity	Create opportunities for those with high referral rates to be identified as Respiratory or PR specialists within the practice.
1.2 Problem solving	Environment, context and resources	At a practice level discuss problems resulting in reduced referral rates and collectively generate and implement local strategies address these and to improve referral.
12.1 Restructuring the physical environment	Environment, context and resources	Make the referral process easier by streamlining access to referral forms or by reducing necessary paperwork.
6.2 Social comparison	Social influences	Draw attention to positive referral rates in other comparative GP practices.
6.3 Information of others’ approval	Social influences	Provide information to teams about the importance practice management place upon maximising PR referral to improve patient self-management of COPD
4.2 Information about antecedents	Knowledge	Provide information about reliable steps that can be put into place to improve chances of referral – such as having previous PR referral data to hand for each patient at their annual COPD check.
5.3 Information about social and environmental consequences	Knowledge	Give information about NHS cost savings following PR referral due to reduced hospitalisation.
1.9 Commitment	Memory attention and decision making	Get staff to agree to a practice goal of increasing PR referral and to give a concrete commitment, eg commit to hand out PR information to all COPD patients they see.

#### Engagement

The most promising engagement interventions had a mean of 6.0 theoretically congruent BCTs, compared to 4.6 in less promising interventions (Tables S18 and S19).

The triangulation between reviews 1 and 2 indicates that engagement interventions are also relatively well-designed from a theoretical perspective to target key influences identified to influence engagement behaviour. Most interventions (13 of 20) responded to barriers around the social interaction between patient and healthcare professional and the importance of social support from friends and family (TDF: Social influences). For example by including components providing social support – such as one-to-one peer support^81^ and patient support offered through a social media led decision aid to support people through the decision to attend PR^94^ (BCT: *Social support unspecified)*. Interventions also commonly provided practical guidance on factors such as how to have a productive conversation with your healthcare team over PR as well as the provision of detailed instructions on practical aspects of COPD self-management^94^ (BCT: *Instruction on how to perform behaviour)* and providing published evidence of the effectiveness of PR to patients^90^ (BCT: *Information on health consequences)* which target the patient's lack of knowledge about COPD, and PR and its value (TDF: Knowledge and Belief in capabilities). Other approaches demonstrated theoretical congruence with barriers to engagement identified in review 1 but were not used in many interventions. For example, supporting participants to develop solutions to address personal barriers to engagement^99^ (BCT: *problem solving*, TDF: Environment, context and resources, used in 3/30 interventions); and visiting patients at home and discussing wider factors influencing COPD and ability to engage in PR, like the need for smoking cessation^105^ (BCT: *Information about antecedents*, TDF: Knowledge, used in 2/20 interventions). (Figure 8). Several theoretically congruent BCTs were infrequently used in intervention components (1/20 interventions), despite theoretical evidence that they will be effective against the influences reported in review 1. Examples include helping patients set their personal goals^86^ (BCT: *Goal setting*) and provision of praise to patients upon achieving good results within the PR session^99^ (BCT: *Social reward*), which can address lack of self-belief in the ability to take part in PR (TDF: Belief in capabilities). Tables S23 and S24 provide further detail (Supplementary File).

Overall, influences in the 4 of the 5 key domains identified as being most relevant for supporting engagement behaviour (TDF: *Belief in Capabilities; Knowledge; Environment context and resources*; and *Social influences)* were relatively well targeted by existing interventions. Barriers concerning the emotional burden of COPD and anxiety about exercising in a group and how this influences engagement with PR were, however, poorly considered in existing interventions and present an opportunity for future intervention development. Possible approaches to address emotional barriers may include supporting participants to the emotional benefits of PR (BCT: *Information about emotional consequences; Body changes*) and assisting them to reconsider being in a group as a positive thing where they will meet others with the same condition and receive social support (BCT: *Framing/reframing*). See Supplementary File for schematic showing the relationship between relevance of barrier (Frequency of coding) and frequency of use of theoretically congruent BCTs (Figure S9).

**Table 10:** Theoretically Congruent BCTs Which Offer Potential for Use in Future Engagement Interventions

BCT	TDF Domain	Example of Potential Operationalisation
11.5 Conserving mental resources	Environment, context and resources	Advise ways to reduce demands on mental resources to allow focus on PR – for example, having additional support with key tasks like shopping during this period
12.2 Restructuring the social environments	Environment, context and resources, social influences	Offer support to engage with others on the waiting list for PR
6.2 Social comparison	Social influences	Give participant information about the number of others who have benefited from taking up the offer of PR in the local area
6.3 Information about other’s approval	Social influences	Send an encouraging letter from the GP when participant has completed the PR assessment and is accepted on to a PR programme
5.5 Anticipated regret	Emotions	Discuss with the patient how they may regret it if they do not learn to successfully self-manage their condition
12.6 Body changes	Emotions	Alert people to the emotional benefits they will expect to experience following PR attendance
13.2 Framing/Re-Framing	Emotions	Use stories from others about the benefits of being in a group to change perceptions about anxiety of being in a group to the group being a positive aspect of PR
15.1 Verbal persuasion about capability	Belief about capabilities	Discuss with participants that the exercises are planned specifically for people with COPD and therefore they will be able to take part without putting themselves in danger
15.3 Focus on past success	Belief about capabilities	Get people to think about times when they have successfully engaged in physical activity in the past
15.4 Self-talk	Belief about capabilities	Encourage people to remind themselves that breathlessness from exercising is not dangerous

Other missed opportunities, where there is evidence of effectiveness to influence the barriers and enablers identified in review 1 for engagement behaviours are listed in Table 10, together with ideas for how the BCTs may be delivered. See Table S25 for further detail.

As a final stage of the analysis, the review of broader system level decisions and actions that may support interventions targeted to address the influences on both referral and engagement behaviours was considered. The interventions reported here are primary service provision interventions. Using the BCW guidance (Table S26) wider system level and policy approaches that are theoretically congruent with the reported influences in review 1 include: Communications (such as awareness raising to healthcare teams and patients via use of print, electronic and broadcast media), Guidelines (such as recommendations to mandate practice), Fiscal measures (such as financial rewards for practices), Regulation (such as establishing rules around opt-out or building PR into routine recommended/mandated care), and Environmental planning (for example, offering PR in more convenient community locations) (see Supplementary File, Tables S27 and S28).

## Discussion

Identifying behavioural influences is a prerequisite for systematic intervention design to improve PR referral and engagement.^18^,^107^ This review presents a synthesis of the evidence on influences on behaviours associated with PR referral and engagement and on interventions developed to address them. Our theoretical congruence analysis indicates that interventions to improve referrals to PR target some of the key influences on referral behaviour well, such as providing social support, developing knowledge and understanding of PR and highlighting the importance of PR referral to their patient care responsibilities. Interventions less frequently contained components that supported practitioners to remember and to streamline the referral process (TDF: Memory, attention and decision making). Similarly, patient engagement interventions were well-targeted to address identified influences on behaviour, such as poor knowledge, lack of self-belief to exercise with COPD and to increase support. Emotional barriers, such as fear and anxiety, were largely unaddressed in existing interventions. There is a paucity of data exploring PR engagement amongst people from minority ethnicities or targeting interventions toward carers and family. Our review outlines strategies to improve intervention targeting and effectiveness and offers a theoretical perspective to explain how interventions reported to be effective are supporting behaviour change. Overall, this work can inform intervention design and policy developments.

The strengths of this analysis are the inclusion of two systematic reviews, one of the qualitative literature and one exploring existing interventions. The associated behavioural analysis provides both a behavioural diagnosis of factors influencing referral to and engagement with PR across a range of geographical settings and a systematic evaluation of the behaviour change “active ingredients” in existing interventions. Adding a cross-cutting strategic analysis to explore theoretical congruence provides a useful insight into where there are further opportunities to adopt innovative strategies. The importance of each TDF domain as an influencer of behaviour was calculated quantitatively, according to frequency. We also supported this by a measure of elaboration to describe the richness of the description. There are some researchers, however, who argue against using quantitative methods in qualitative research and there are limitations to evaluating importance based on frequency in qualitative analysis.^108^ The limitations include low representation of literature concerning ethnic communities who are minoritised in European, North American and Australasia healthcare settings. This limits transferability to people in communities acknowledged to experience inequities in access to PR. Similarly, the majority of papers come from affluent healthcare settings limiting generalisability to other settings. Soft measures of intervention effectiveness were used as quantitative meta-analysis was not feasible with the heterogeneity of interventions included. It is encouraging, however, that interventions recognised as most promising were also identified in previous reviews.^17^,^18^ The TIDieR framework is recommended for describing interventions in publications.^109^ Few publications reported their interventions thoroughly, which may mean other BCTs may have been employed but the detail not reported. As promise ratios could not be calculated for all BCTs, they were grouped by type, limiting the granularity of understanding of the most effective BCTs. BCTs used in interventions associated with PR delivery are less likely to show theoretical congruence, as in step 1 papers did not identify barriers and enablers to PR delivery, independent of referral.

### Improving Process and Resource-Led Factors

Practical and resource-related barriers for referral to PR by HCPs, such as lack of time, lack of standardised systems and appropriate promotional materials, were also reported in the review by Cox et al^8^ Similarly, facilitative factors such as streamlined procedures and PR coordinators have also been previously identified.^8^,^11^,^110^ Previous reviews also support evidence of effectiveness of interventions offering streamlined PR referral procedures such as computerised templates^106^ and including automatic referral in discharge bundles.^17^,^18^,^101^ These interventions target TDF domains of Memory, attention and decision making and Environment context and resources using theoretically congruent BCTs such as Environmental restructuring, Conserving mental resources and Prompts and Cues. The current degree of stress and burnout in primary care in the UK and other high-income settings is well reported^111^ and interventions to improve referral need to be considered in this context, making interventions that streamline procedures attractive. In the UK NHS^112^ and other healthcare services globally,^113^ a focus on streamlining and automating referral into specialist and community services, especially through digital means,^114^ does present an opportunity to address these barriers and this review suggests these approaches are likely to be effective in supporting positive change in PR referral. Similarly, organising management of COPD and other respiratory conditions in integrated community teams in the UK is likely to continue to improve integration with, and referral to, PR services.^115^

### Overcoming Practical Patient Barriers

It is interesting to note that interventions targeting patient engagement showed less promise than those focussing on referral. Whilst progress is needed to support both activities, a particular focus on improving patient engagement is warranted. Within the TDF domain of Environment, context and resources patient barriers were associated largely with the practical challenges of managing competing demands on their time, challenges with getting to venues as well as costs associated with attendance. Problems were understandably exacerbated when dealing with multiple comorbidities and severity of COPD. Supporting this, Hayton et al have suggested challenges with transport are exacerbated for patients needing ambulatory oxygen.^116^ Whilst NHS transport is available, our focus groups carried out with healthcare professionals as part of the UPTURN study (unpublished data) suggest that wait times associated with NHS transport can be difficult for PR participants, as PR services are often provided in community venues that are unattended after PR delivery but where participants potentially face a long wait for their booked transportation. A review of strategies, offered by services to overcome transport barriers to PR sessions in the USA, included the provision of free parking, parking vouchers, charity-delivered car service, ride-sharing and direct provision of transport but the efficacy of such strategies remains uncertain.^117^ Our findings align with previous reviews suggesting that interventions focusing on tele-PR or home-based service provision can address recognised barriers to engagement.^8^,^11^ These interventions are theoretically congruent to support environmental and contextual barriers and incorporate the BCT Restructuring the Physical environment, amongst others. Cochrane review data supports the effectiveness of remote PR services in delivering meaningful clinical outcomes, where face to face PR is not feasible.^24^ However, the current positioning statement, even with a recent update to consider 4 new trials, still promotes face to face PR as the preferred gold standard.^118^

### The Influence of Lack of Knowledge and Awareness of PR

It is perhaps surprising that lack of knowledge of PR and its benefits, suggested in our review and others,^11^ is a limiting factor for both healthcare professionals and patients, given the robust evidence supporting the effectiveness of PR.^6^,^119^ This suggests the value of education and awareness building. Amongst the 7 referral interventions using educational content, the 3 showing most promise in our review and others^17^,^18^ tended to include a wider range of BCTs than just those specifically related to knowledge about PR, such as *Social Support*. Our data therefore offers insight to suggest that effectiveness of these interventions may in part be influenced by the associated social support of peers. Similarly, for targeting patients, the most promising intervention^94^ was the only one to incorporate BCTs related to social support and it also included 9 theoretically congruent BCTs targeted at domains associated with motivation and opportunity (compared to 4 in the less promising interventions). While it may not be obvious to consider the importance of social support in the context of referral, these findings are unsurprising as it is well reported that whilst knowledge is necessary, knowledge alone does not lead to behaviour change.^120^ Models such as COM-B incorporate the consensus opinion that for behaviour to occur consideration of motivational factors and social and environmental constraints are also necessary.^121^

### Supporting the Emotional Health of Patients

The main difference between this analysis and the review carried out by Cox et al,^8^ which this review updates, is the prominence of themes related to emotions. The emotional burden of COPD and associated barriers to PR engagement (eg fear and anxiety) seem poorly considered in existing interventions People describe being “emotionally and existentially disturbed” as a result of living with COPD^122^ They feel vulnerable, ashamed and stigmatised,^122^ living in constant fear of breathlessness and increasing feelings of loneliness.^123^,^124^ Negative emotions have been shown to impact on self-care strategies and engagement with healthcare in people living with chronic conditions^125^ Our analysis indicates an opportunity to target improvements in emotional support for people living with COPD, potentially increasing both uptake and adherence to PR Potential BCTs identified include, *Information about emotional consequences* (eg telling patients about the value of PR in supporting mental health and anxiety); *Reducing negative emotions* (eg give advice to patients to support their management of stress and anxiety, to support their capacity to attend PR); and *Framing, re-framing* (eg supporting patients to see the positive value of improving their fitness and ultimately reduce breathlessness to reduce their fear of exercise-induced breathlessness).

### Interaction Between Patient and HCP

Our data highlights the importance of a positive patient-healthcare social interaction during the referral conversation to support patient engagement with PR Active listening, demonstrating genuine empathy, establishing trust, and empowering shared decision-making are all key to a successful PR referral conversation, according to a recent Australian study.^126^ It is also important to patients that healthcare professionals instil hope about the value of PR, allay patient fears and use patient friendly language, including alternatives to “Pulmonary Rehabilitation”.^126^ Of the interventions included here, none specifically target patient-healthcare communication. This area offers potential, although the supportive conversation about PR may well need to take place post-referral within PR providers or wider integrated teams, rather than time-pressured primary care settings.

### Increasing Accessibility and Engagement Amongst People from Minority Ethnicities

In high income countries, both PR referral and engagement rates are lower amongst people from culturally and linguistically diverse communities^127–129^ This review highlights the paucity of data to understand pertinent barriers to engagement amongst people from minority ethnicities and guide how we develop culturally salient interventions to support these patient groups. A recent published review exploring barriers and enablers to a range of COPD treatment interventions amongst people from minority ethnicities indicates the importance of a positive healthcare interaction, provision of social support, delivery in appropriate language and creating a feeling of community belonging, were all important to support engagement.^130^ Additionally, cultural beliefs and commitments to religion, family and work were factors that needed to be considered to improve uptake.^130^

### Recommendations for Intervention Development and Practice

#### Service Provision

1. For referral to PR, this review highlights the value of interventions aimed at developing knowledge amongst healthcare professionals (perhaps also offering the opportunity for peer social support and social comparison), streamlining referral processes, and developing opportunities for a supportive PR conversation between patients and healthcare teams.
2. Recognition of and support to address emotional barriers may enhance patient motivation to engage.
3. Well-reported logistical and practical barriers for patients highlight the likely continued appeal of home-based and tele-PR which support behaviour change by addressing environmental barriers.
4. There is opportunity to consider interventions that engage the family in the provision of social support for patients.

#### Systems-Level Recommendations

1. General public health communications strategies to communicate the purpose and value of PR, to raise the general level of knowledge of COPD patients and healthcare professionals.
2. Guidelines and regulations to support and mandate automatic referral to PR within primary and secondary care, as well offering fiscal reward to practices may support service provision.
3. Support opportunities to enhance the PR conversation between patients and HCPs. This may include, for example, continued expansion of community integrated respiratory care teams, supporting PR providers to discuss PR in more depth following referral or supporting primary care staff with appropriate training and resources.
4. To improve physical accessibility of PR, expanding venue choice and improving infrastructure. This may include support for community services to develop partnerships with the VCSE sector to support people’s health at a local level, as recommended by the King’s fund.^131^

#### Research Gaps

1. Conduct further research to understand the barriers to engagement for people from minority ethnicities and explore how engagement can be supported in a culturally salient manner.

## Conclusions

There are continuing calls to provide recommendations to improve the uptake of PR globally, to make recommendations for evidence-based policy^132^ and to develop theoretical understanding of what makes interventions successful.^17^ This study develops our theoretical understanding of how the most promising interventions may support PR referral and engagement behaviours. In addition, it highlights potential to develop interventions to engage family support and the need to explore and respond to the needs of people facing cultural and linguistic barriers to respiratory care.

## Data Availability

Protocols are published in Prospero. No other additional data is available.

## Abbreviations

BCT, Behaviour Change Technique; BCTT, Behaviour Change Technique Taxonomy; BCW, Behaviour Change Wheel; COPD, Chronic obstructive pulmonary disease; NHS, National Health Service (UK); PR, Pulmonary rehabilitation; PRatio, Promise ratio; TDF, Theoretical Domains Framework; TaTT, Theories and Techniques Tool; USA, United States of America.
